# Temporary threshold shift after noise exposure in hypobaric hypoxia at high altitude: results of the ADEMED expedition 2011

**DOI:** 10.1007/s00420-021-01715-w

**Published:** 2021-05-22

**Authors:** K. Fehrenbacher, C. Apel, D. Bertsch, M. S. van der Giet, S. van der Giet, M. Grass, C. Gschwandtl, N. Heussen, N. Hundt, C. Kühn, A. Morrison, M. Müller-Ost, M. Müller-Tarpet, S. Porath, J. Risse, S. Schmitz, V. Schöffl, L. Timmermann, K. Wernitz, T. Küpper

**Affiliations:** 1 Institute of Occupational and Social Medicine, RWTH Aachen Technical University, Pauwelsstr. 30, 52074 Aachen, Germany; 2 Department of Dental Preservation, Parodontology and Preventive Dentistry, RWTH Aachen Technical University, Aachen, Germany; 3 Department of Cardiology, Catholic Hospital Marienhof, Koblenz, Germany; 4 Department of Medical Statistics, RWTH Aachen Technical University, Aachen, Germany; 5 Medical School, Sigmund Freud Private University, Vienna, Austria; 6 Royal Free London NHS Foundation Trust, London, UK; 7 Department of Technical Acoustics, RWTH Aachen University, Aachen, Germany; 8 Medical Commission of the Union Internationale des Associations d’Alpinisme (UIAA MedCom), Bern, Switzerland; 9 Department of Sports Medicine-Sports Orthopaedics, Klinikum Bamberg, Bamberg, Germany; 10 Department of Trauma Surgery, Friedrich Alexander University Erlangen-Nuremberg, Erlangen, Germany

**Keywords:** Noise, Hearing loss, Hypoxia, High altitude, Acclimatization, Temporary threshold shift

## Abstract

**Objectives:**

To evaluate whether there is an increased risk for noise-induced hearing loss at high altitude rsp. in hypobaric hypoxia.

**Methods:**

Thirteen volunteers got standard audiometry at 125, 250, 500, 750, 1000, 1500, 2000, 3000, 4000, 6000, and 8000 Hz before and after 10 min of white noise at 90 dB. The system was calibrated for the respective altitude. Measurements were performed at Kathmandu (1400 m) and at Gorak Shep (5300 m) (Solo Khumbu/Nepal) after 10 days of acclimatization while on trek. Temporary threshold shift (TTS) was analyzed by descriptive statistics and by factor analysis.

**Results:**

TTS is significantly more pronounced at high altitudes. Acclimatization does not provide any protection of the inner ear, although it increases arterial oxygen saturation.

**Conclusion:**

The thresholds beyond which noise protection is recommended (> 80 dB) or necessary (> 85 dB) are not sufficient at high altitudes. We suggest providing protective devices above an altitude of 1500 m (“ear threshold altitude”) when noise level is higher than 75 dB and using them definitively above 80 dB. This takes the individual reaction on hypobaric hypoxia at high altitude into account.

## Introduction

Traditionally noise exposure of employees in a hypobaric environment was a potential risk for a limited collective, for example for pilots, alpine rescue personnel—especially when helicopters are in us—and a few construction or maintenance workers at construction sites at high altitude. Recently the number of noise-exposed persons increased significantly for at least two reasons: there is more and more international industry with numerous projects and construction sites all over the world including the world’s high altitude regions as well as the isobaric atmosphere.

Although many papers have been published about the noise exposure of helicopter rescue personnel (Kupper et al. 2004; Kupper et al. 2013) or hearing loss of pilots [detailed survey in Kupper et al. (2013)], the combined effect of noise and hypoxia was rarely discussed. Typically alpine rescue operations take place at altitudes between 2500 and 4000 m (Kupper 2006). At these altitudes, the oxygen pressure is reduced by a third (Ernsting and King 1994; Kupper et al. 2010). There are also numerous sites where employees work at even higher altitude rsp. lower oxygen pressure, e.g. the astronomers of the European south observatorium (Chajnantor-Plateau, Atacama desert, 5039 m), military personnel (especially special forces), the mines in the Andes at ~ 4000–5000 m (Richalet et al. 2002), or at construction sites for power plants in Tibet at 4050 m (Küpper and Storch 2013).

Hearing is an active, energy-consuming process and this energy is provided inside the cochlea over a relatively long diffusion distance. By this, the temporary threshold shift (TTS) after noise exposure is interpreted as an “energetic exhaustion” of the cochlear cells. In hypoxic conditions, this may become critical. Experiments with animals have shown that there is a significant reduction of cochlear perfusion after exposure to 85 dB (A) for 6 h (Attanasio et al. 2001). This may be interpreted as indirect proof of reduced oxygen delivery to the inner ear. Other investigations found such effects at higher levels only [> 100 dB (A)], but then linearly correlated it to increasing sound levels and to the decrease of perilymphatic oxygen partial pressure (Lamm and Arnold 1996). Both effects lasted for at least 1 h after the noise exposure was ended and a complete recovery was reached after a much longer period (3 h). Attitas et al. showed in their animal model that a TTS occurred after an isobaric exposure to 6% oxygen (Attias et al. 1990), which corresponds to an altitude of about 10,500 m (Ernsting and King 1994; Kupper et al. 2010). The finding that acclimatized animals showed significantly lesser hearing impairment supports the thesis of the combined effect of noise and hypoxia (Berndt et al. 1978).

The results of hearing loss of pilots are somewhat contradictory. As discussed later we are sure that this is caused because physical effects of noise in the air of lower density and therefore lower mechanical energy transmission were not taken into account by all previous studies.

To our best knowledge, there is only one study which aims to investigate the combined effect of noise and hypoxic environment in humans. Non-acclimatized probands were investigated at a corresponding altitude of about 4500–5500 m. They showed significant TTSs (Fowler and Grant 2000). However, systematic investigations are missing although these effects indicate that the thresholds given for safe work in noisy environments [e.g. Anonymous (2003), Anonymous (2004)] may not be safe for persons working in hypoxic conditions. Therefore we investigated hearing and TTS in a collective going to high altitude. In contrast to earlier studies, the mechanical effect of thin air at high altitude was included in the actual investigation.

## Methods

The collective were healthy men (four) and women (nine) who were participants of the scientific ADEMED expedition 2011 to the Everest region (Nepal) and volunteered to participate [age 22–29 years, except from no.12 (52 years)]. Nobody had known diseases or problems with their ears like impaired hearing (e.g. noise-induced hearing loss). Nobody took any drug against acute mountain sickness or others that might interfere with the cardiocirculatory system or with peripheral perfusion. The study was approved by the Ethical Commission at the RWTH Aachen University Hospital (No. Ek 196/11).

The auditory threshold of every participant was measured by standard audiometry before and after standardized noise exposure. For audiometry Oscilla USB 350B was used (Voss Medizintechnik GmbH, Hamburg, Germany) with the software Oscilla^®^ AudioConsole V3.8.8 (Inmedico A/S, Skejby, Danmark). In random order (frequency and side of ears) sounds at 125, 250, 500, 750, 1000, 1500, 2000, 3000, 4000, 6000 and 8000 Hz were played with a sound level stepwise increasing by +5 dB until the proband indicated the perception of sound.

**Fig. 1:**
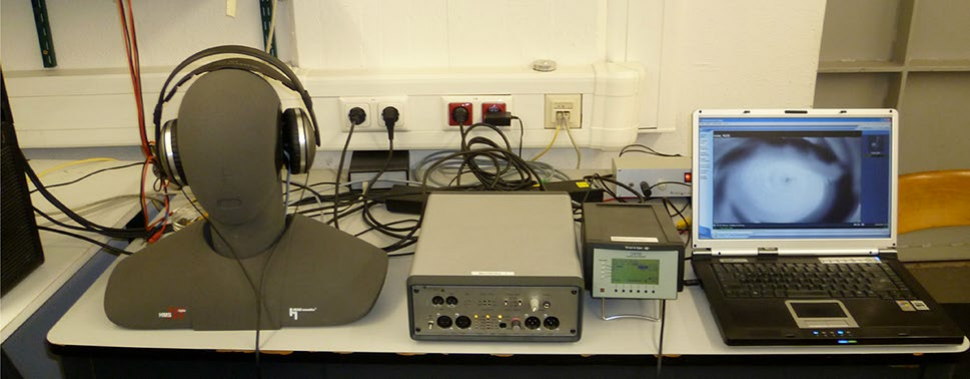
Calibration system for Sennheiser earphones for white noise exposure

To induce a TTS under standardized conditions every hair cell of the ear had to be exposed to the same amount. Therefore a calibrated white noise of 90 dB (A) was used for 10 min as noise exposure between both audiometries at each altitude. The systems used were a laptop and Sennheiser earphones (type HD 250 linear, Sennheiser electronic, Wedemark, Germany) that were calibrated using a dummy head microphone (Fig. 1) by the Institute for Technical Acoustics, RWTH Aachen University.

To minimize the influence of any possible noise exposure during the day all measurements were undertaken in the early morning. By this, another effect that otherwise might have biased has been excluded: the influence of physical exercise was shown to cause a greater TTS under noise exposure than without activity (Lindgren and Axelsson 1988; Miani et al. 1996). Since SaO_2_ shows a circadian rhythm (Tannheimer and van der Sperk 2017; Cristancho and Riveros 2016) this bias was excluded as much as possible by measuring at the same daytime.

**Fig. 2:**
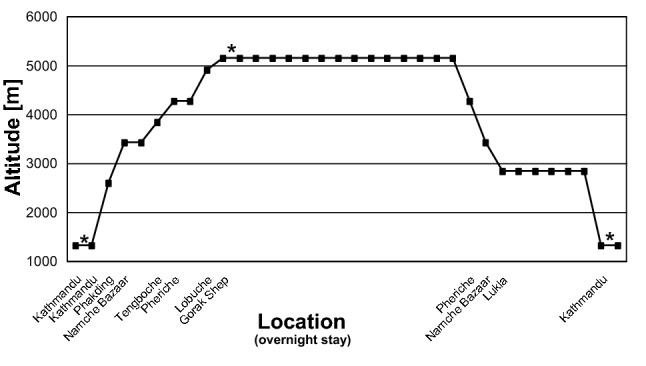
Altitude rsp. acclimatization profile of the collective. The locations of measurements are indicated by asterisks

To evaluate the effect of hypobaric hypoxia the measurements were performed at 1400 m above sea level (Kathmandu) and at 5300 m above sea level (Ghorak Shep, 5300 m). After altitude exposure, another measurement was conducted at Kathmandu to determine whether acclimatization might have a positive effect to prevent a TTS. The altitude rsp. acclimatization profile is shown in Fig. 2. To exclude environmental noise all measurements were performed in a quiet room of the lodges after the guests had left.

### Adjustment of noise exposure for altitude

For mechanical reasons (reduced air density at altitude), the sound level of the white noise has to be adjusted according to the altitude of the respective measurement. To create a noise exposure at different altitudes $h$ with the same sound pressure, the ear and the headphone have to be considered to build an acoustic chamber with volume $V_{0}$ and a membrane surface of the headphone $S$. The relation between deflection of the membrane $x$ caused by the electrical audio signal of the laptop’s sound card AD converter and the sound pressure $V_{0}$ in the chamber is given by

1 $$ p = \frac{{\rho_{{0}} c^{{2}} }}{{V_{{0}} }}Sx , $$

where $\rho_{0}$ is the mass density and $c$ the speed of sound of the medium, i.e. air. Here, the assumption is made that all dimensions are small with respect to the wavelength and that the chamber can be considered sonically hard.

Both, mass density and speed of sound depend on temperature $T$. However, $\rho_{0} \propto \frac{1}{T}$ and $c^{2} \propto T$. Therefore, the product $\rho_{0} c^{2}$ does not depend on temperature. According to the ideal gas law and assuming pressure and mass density perturbations to be much smaller than the reference values,

2 $$ \rho_{0} c^{2} = \kappa p_{0} {,} $$

where $\kappa = 1.4$ is the adiabatic exponent of air and $p_{0}$ is the surrounding static air pressure, which depends on the altitude according to the well-known barometric formulae

3 $$ p_{0} \left( h \right) = 1013.25\left( {1 - \frac{{0.0065\frac{K}{m}h}}{28815 K}} \right)^{5255} hPa. $$

From (2) and (1), it can be seen that the same input deflection of the headphone membrane causes smaller sound pressure at higher altitudes. Substituting (3) in (2) and (1) and taking the logarithm, the difference in sound power level, depending on altitude can be obtained

4 $$ {\Delta }L_{p} \left( h \right) = {\text{log}}_{{{10}}} \left( {1 - \frac{h}{{44,331{\text{m}}}}} \right) \cdot 105.1 {\text{dB}}{.} $$

**Fig. 3:**
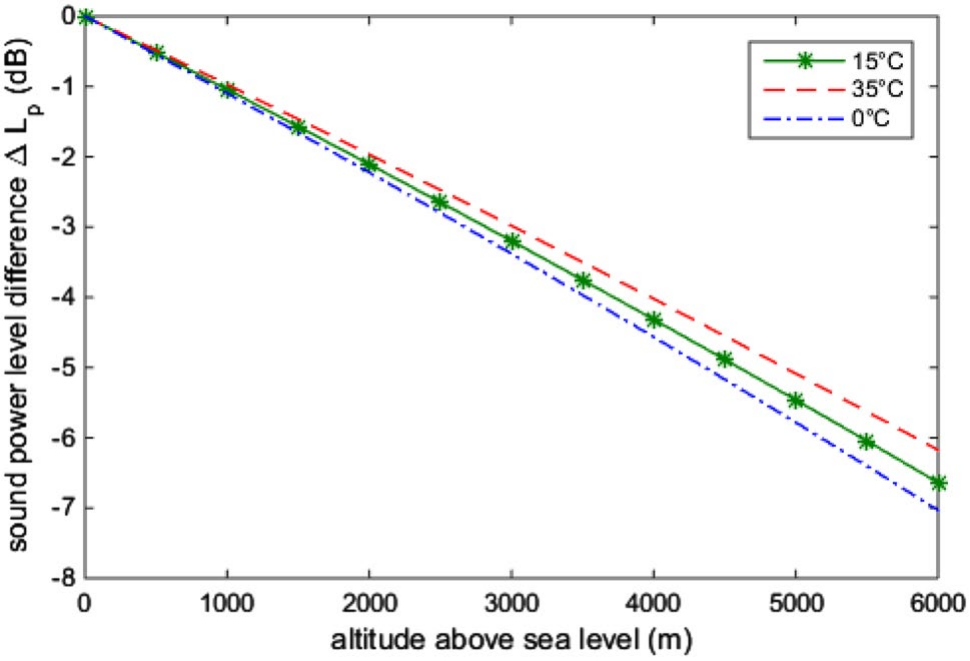
Sound pressure level difference depending on altitude and temperature

Figure 3 shows this relation. It means that the effect of a headphone signal on the sound pressure level is reduced at higher altitudes. As can be seen, the relation is approximately linear in the altitude range of interest. The sound pressure decreases by approximately 1 dB per 1000 m. To compensate for this reduced sound pressure level, the white noise exposure has been increased by 1 dB per 1000 m during the experiments.

Strictly speaking, the barometric formula (3) is only valid for 15 °C ambient temperature. Even under the worst conditions during the measurements in the field, the air can still be assumed to be in a range from 0 to 35 °C. Figure 3 shows that the influence of the temperature is significantly smaller than the reduction due to altitude.

### Statistical analysis

The primary outcome TTS was calculated as the difference between the auditory threshold [dB (A)] before and after noise exposition for each setting. The settings for each of the 13 participants are defined by 11 different frequencies in the left as well as in the right ear. Measurements are taken at three different conditions resulting from combinations of low altitude and acclimatization i.e. 1400 m non-acclimatized, 5300 m, and 1400 m acclimatized.

Comparisons of the different conditions with respect to TTS were performed with a linear mixed-effects model with random intercept and variance components as covariance structure. The condition was modelled as a fixed effect, in addition the auditory threshold [dB (A)] before noise exposure was included in the model to adjust for differences in baseline measurements. Moreover, the side of measurement (left or right ear) and the frequency were modelled to reflect the repeated measurements within one participant.

Pairwise comparisons between different conditions were evaluated by corresponding linear contrasts. Model assumptions and model fit were checked by visual inspection of the residuals, and the measures of influence diagnostics. Observations with strong influence on estimates and their precision were removed from the respective analysis and evaluated in a sensitivity analysis.

Missing values were taken into account by a likelihood-based approach within the framework of mixed linear models with the assumptions that missing values occur at random. For all comparisons the significance level was set at 5%; due to the explorative nature of this study, no adjustment was made to the significance level. Results are reported as estimated means and standard errors (SE), two-sided *p *values were accompanied by values of the test statistic (*t*) and degrees of freedom (*DF*). In addition, 95% confidence intervals (CI) for the differences in mean TTS were provided. Boxplots were used to visualize the distribution of the data. All analyses were performed with the SAS version 9.4 (PROC MIXED; SAS Institute Inc., NC, USA).

As discussed below proband Number 12 was excluded later from evaluation because it turned out that he suffered from a previously not known noise-induced hearing loss.

## Results

**Fig. 4:**
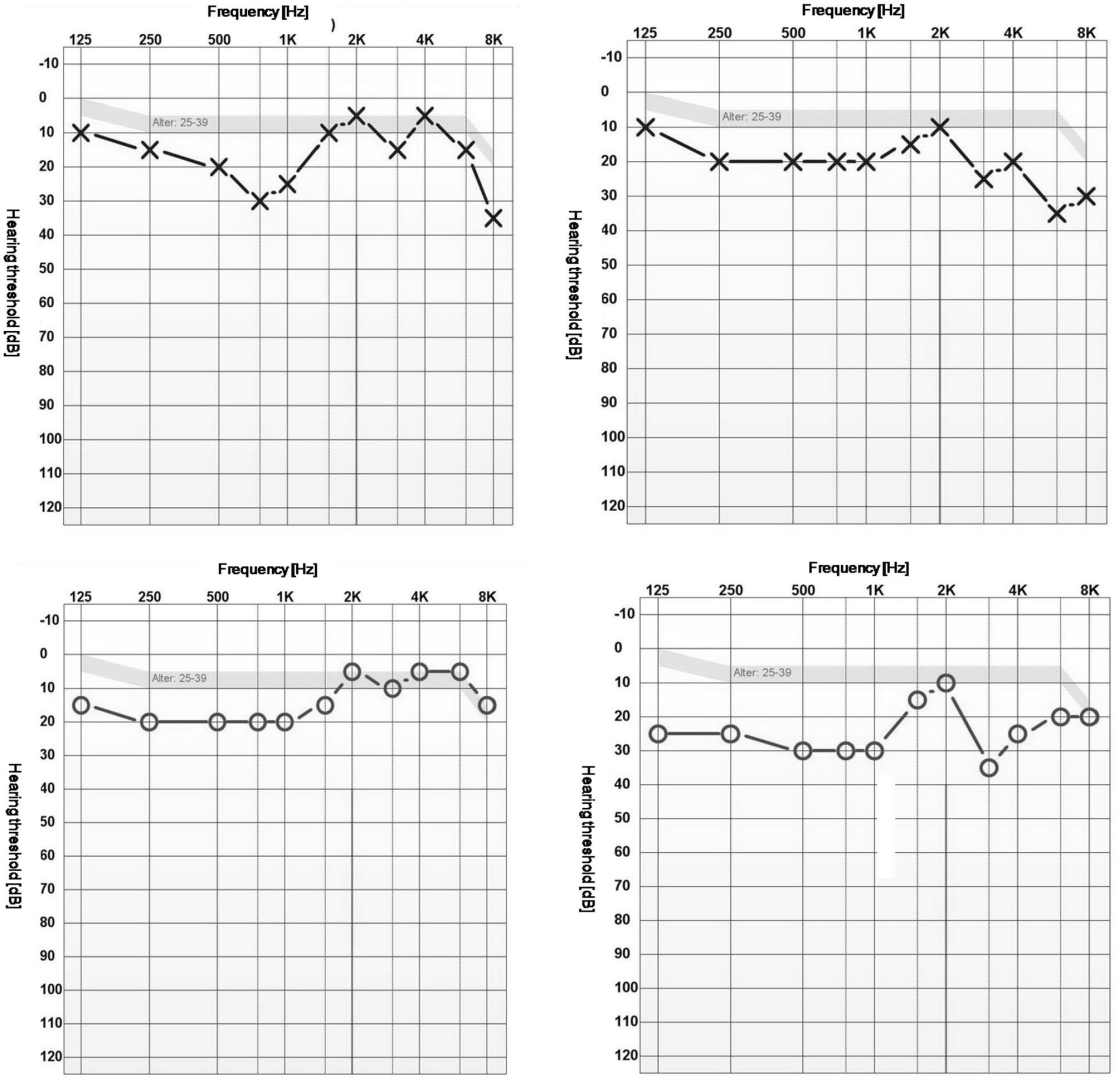
Example of an audiogram at 5300 m (Gorak Shep) before (left column) and after noise exposure (right column). Upper graphs: left ear; lower graphs: right ear

**Fig. 5:**
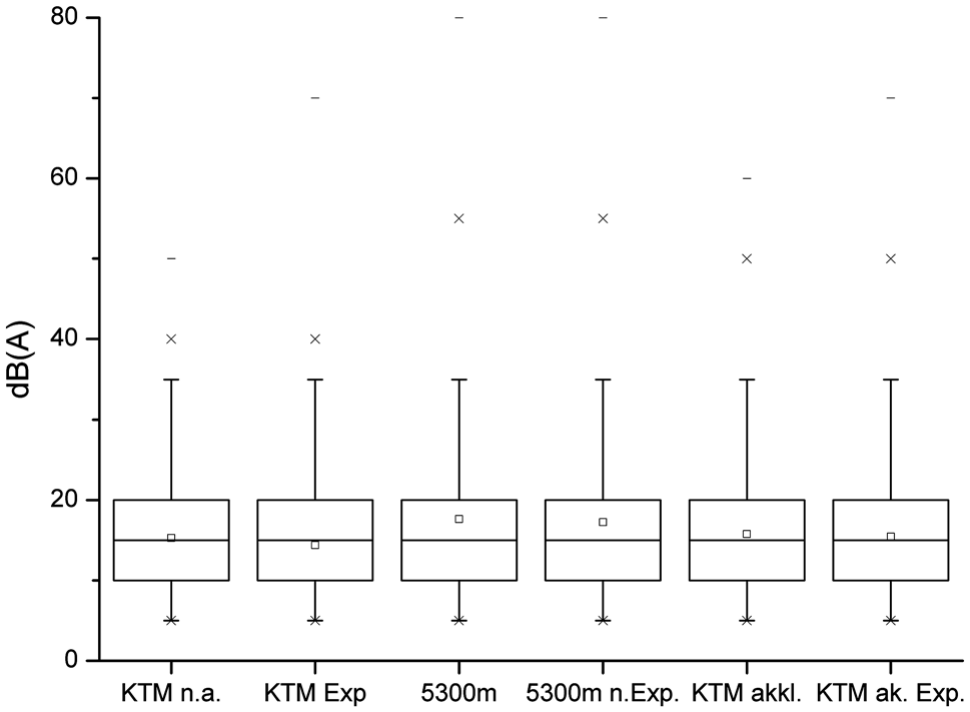
Raw data before and after the exposition and non-acclimatized vs. acclimatization (*KTM n.a.* Kathmandu, not acclimatized, before noise exposure; *KTM Exp* Kathmandu, after white noise exposure; 5300 m: Gorak Shep, before noise exposure; 5300 m; *n. Exp* Gorak Shep, after white noise exposure; *KTM akkl.* Kathmandu, after return from altitude, without noise exposure; *KTM ak. Exp.* Kathmandu, after return from altitude and after white noise exposure

The auditory threshold averaged overall measurements with corresponding standard deviations are summarized in Table 1 for each condition before and after noise exposure. An example of audiograms before and after exposure at 5300 m (Gorak Shep) is given in Fig. 4. Mean auditory thresholds are comparable between both measurements within low and extreme altitudes (Fig. 5).

**Table 1:** Auditory threshold at the different measuring locations

	Mean (standard deviation) of auditory threshold [dB (A)]	
Condition	Before noise exposure	After noise exposure
KTM (1800 m, non-acclimatized)	15.3 (8.1)	14.4 (8.7)
GS (5300 m)	17.6 (10.5)	17.2 (9.7)
KTM (1800 m, acclimatized)	15.8 (8.7)	15.2 (9.0)

**Fig. 6:**
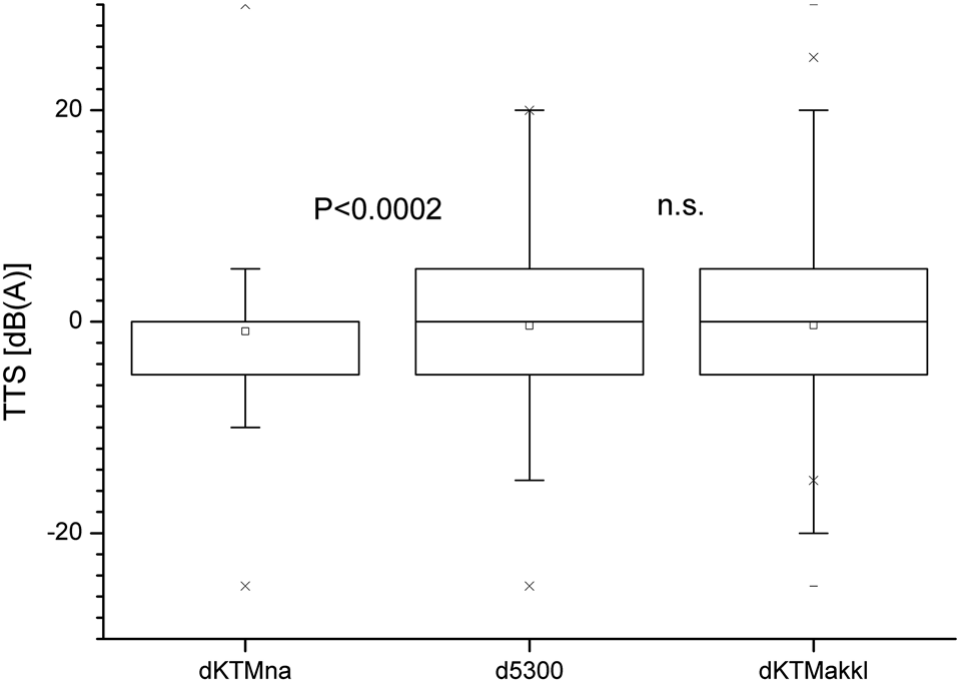
Temporary threshold shift (TTS), all participants (*dKTMna* shift at Kathmandu without acclimatization; *d5300* shift at Ghorak Shep; *dKTMakkl* shift at Kathmandu after high altitude acclimatization)

Pairwise comparisons of the mean temporary threshold shift between the three conditions show a significant difference between Kathmandu before acclimatization compared to Ghorak Shep { − 1.3 (0.5) vs. 0.2 (0.5) dB (A), *p* = 0.0048, *t* = − 2.83, *DF* = 775, 95% CI [ − 2.48 dB (A); − 0.45 dB (A)]}. No significant difference in mean TTS was found between Kathmandu after acclimatization compared to Ghorak Shep { − 0.9 (0.5) vs. 0.2 (0.5) dB (A), *p* = 0.0541, *t* = 1.93, *DF* = 790, 95% CI [ − 0.019 dB (A); 2.13 dB (A)]}. In addition, the comparison of mean TTS between Kathmandu before and after acclimatization leads to a non-significant difference { − 1.3 (0.5) vs.  − 0.9 (0.5) dB (A), *p* = 0.4518, *t* =  − 0.75, *DF* = 791, 95% CI [ − 1.48 dB (A); 0.66 dB (A)]} (Fig. 6).

Visual inspection of the influence statistic (Cook’s D) showed a strong influence of the measurements of participant no. 12. This observation was associated with an audiometric finding: participant no.12 had a pre-existing C5-dip which probably was the effect of previous occupational noise exposure while working as helicopter emergency medical services (HEMS) crew member of an alpine rescue service for 6 years.

A sensitivity analysis intensified the trend, which was observed in the primary analysis. The comparison of mean TTS without data from the participant no. 12 showed now a statistically significant difference between Kathmandu after acclimatization compared to Ghorak Shep { − 1.0 (0.6) vs. 0.6 (0.5) dB (A), *p* = 0.0014, *t* = 3.21, *DF* = 7220, 95% CI [0.63 dB (A); 2.60 dB (A)]}. The comparison between Kathmandu before acclimatization and Ghorak Shep revealed again a significant difference as well { − 1.5 (0.5) vs. 0.6 (0.5) dB (A), *p* < 0.0001, *t* = − 4.52, *DF* = 711, 95% CI [− 3.05; − 1.20]}. The comparison between Kathmandu before and after acclimatization showed a non-significant result { − 1.5 (0.5) vs. − 1.0 (0.6) dB (A), *p* = 3064, *t* = − 1.02, *DF* = 722, 95% CI [ − 1.50; 0.47]}.

## Discussion

The main finding of the actual study is that the inner ear is more sensitive when exposed to noise at altitude (hypobaric hypoxia) compared to low altitude. Obviously, acclimatization does not balance this increased sensitivity. Therefore special care is needed when people are exposed to noise in such conditions.

Hearing loss is one of the most common occupational diseases (Catlin 1986). The extent of hearing loss is based on the magnitude of sound pressure level, the time of exposition, and the individual disposition or risk factors. The mechanism is commonly hypothesized as follows: when hearing cells are exposed to noise, ATP will be released into the stria vascularis cochleae to support the function of these cells. The important role of ATP in the process of hearing indicates the need and the significance of energy in the course of events. When the inner ear is exposed to noise over a certain time and above a certain noise level an imbalance between demand and use of energy will occur. An energetic exhaustion or tiring of hair cells is the consequence which results in a lower sensitivity of the cells, the so-called “shift of auditory threshold” or “temporary threshold shift” (TTS). Accordingly, the TTS seems to be the consequence of depletion of energy by high exposure to noise (Munoz et al. 2001). Should the noise exposure continue without a sufficient recovery time to built up an adequate amount of ATP again, a breakdown of cellular integrity and afterwards a degeneration of related nerves is imminent. Then, an irreversible shift will have occurred, the so-called “permanent threshold shift” (PTS) (Chen et al. 2007).

To understand the additional impact of hypoxia on the hearing cells it is important to take into account the problem of oxygen supply to those cells. According to Morgenstern et al. oxygen is transported to the inner ear through the round window (Morgenstern and Kessler 1978). Though Morgenstern et al. also published, that the oxygen supply of the inner ear is accomplished by diffusion over the round window and the blood (Morgenstern and Kessler 1978) there are other studies which state that the only way of oxygen supply is by diffusion through the endolymph (Gaudin 1972). With this distance of diffusion, it is probable that the oxygen supply of the inner ear is limited and at risk to become insufficient if there is less oxygen pressure in the inspired air or arterial oxygen saturation is low. Then oxygen supply of the inner hearing cells becomes insufficient, the functionality of those cells will be reduced and the cellular integrity endangered. Therefore, temporary damage by noise exposure might occur earlier in a hypoxic than in a normoxic environment. Since TTS is an indicator of temporary metabolic exhaustion of the inner hearing cells it is reasonable to conclude that if the TTS occurs earlier in hypoxic height, the PTS will also occur earlier.

This hypothesis is supported by some results which were published over 5 decades: Rudmose et al. performed audiometric measurements in a hypobaric chamber at sea level and at 35,000 ft (10,668 m) (Rudmose et al. 1948). The subjects were non-acclimatized and there was no noise exposure. This setting caused a mean TTS of 2.5 dB. In 1992 Carlile’s group published two papers: after acute exposure of humans to hypoxia which caused a decrease of arterial oxygen saturation (SaO_2_) to 75–85% there was a significant prolonged latency of the V wave which corresponded to a TTS of about 5% (Carlile et al. 1992). The authors didn’t give details but based on our data the exposure was comparable to 5000–5500 m, which is a similar altitude to those of our site at Gorak Shep. The second study, also by evoked response technique, was performed at sea level, at 500 m, and at 4370 m (Carlile and Paterson 1992). At 3500 m SaO_2_ was 86.5% which caused a TTS of 9.1 dB. Interestingly this shift was not found anymore after 72 h at altitude and a further ascent to 4370 m did not result in another TTS, although SaO_2_ was 82.5% only. The authors concluded that acute exposure causes TTS but that the ear is able to adapt to these conditions. The problem of both studies is the very small sample size and therefore a very limited power of both investigations.

The lower density of the air at high altitude will influence the stimulus that reaches the inner ear (Rudmose et al. 1948; McAnally et al. 2003). McAlly et al. tried to exclude this bias and reported a mean TTS of 2.5 dB for all frequencies measured. Chen investigated the combined effect of noise and hypoxia morphologically (Chen 2002). He found a linear correlation between the damage of hearing cells and increasing hypoxia. This finding would support our hypothesis.

Of special interest for our study are the results of Singh et al. (2004) for two reasons: 1. they disagree with those of Carlile et al. (1992) and 2. their study was performed at a comparable altitude to ours and also with acclimatized subjects. Singh et al. also found a significant prolonged latency of the waves I, III, and V which indicates a significant TTS. Both, Singh’s and Carlile’s study indicate that the ear obviously is more sensitive against hypoxia than previously expected.

Our preliminary data show that TTS after noise exposure is more pronounced in hypobaric hypoxic environments at high altitude than at low altitude or even at sea level. This was significant even though there was a slight variance between the individuals. This indicates that the auditory system is more at risk in hypobaric hypoxia.

A possible explanation for the higher vulnerability of the inner ear in hypoxic environment is the fact, that the cells of the inner ear get oxygen through diffusion, as mentioned above (Gaudin 1972). Diffusion takes place mainly from the vessels in the limbus spiralis and from the perilymph of the scala tympani (Vosteen 1970). Long diffusion distance and reduced oxygen saturation in the capillary blood are both factors which worsen the situation of the cochlear cells.

Based on this “oxygen/energy hypothesis” we assumed that acclimatization has a positive effect on the resistance of inner hearing cells regarding noise exposure in hypoxia. According to Serebrovskaya (Serebrovskaya 2002) adaptation mechanisms caused by intermittent hypoxic training—which has less acclimatization effect than 24 h exposure for several days (Wille et al. 2012)—stimulated “antioxidant defense mechanisms”, “cellular membranes become more stable” and “oxygen transport in tissues is improved”—all those are mechanisms which should also improve the inner ear’s resistance against oxygen depletion. Other studies hinted at an acclimatization effect on the auditory system that reduces the TTS in hypobaric hypoxia (Carlile and Paterson 1992).

To our best knowledge, the effect of high-altitude acclimatization on TTS after return to lower altitude (or even sea level, which was impossible in this study) was never investigated before. We hypothesised that a significant effect should be detectable with less TTS after noise exposure in acclimatized persons. However, considering the data from 12 participants (excluding proband no.12 because of a pre-existing noise-induced hearing loss) there was a significant lower TTS after return to Kathmandu compared to Ghorak Shep. But comparing the TTS at Kathmandu before and after acclimatization, no significant reduction or elevation of TTS was detectable. We, therefore, conclude that the difference after returning from Ghorak Shep was probably mainly effected by the higher *p*O_2_ at Kathmandu and that acclimatisation does not protect the inner ear against noise. This may be explained by several factors: first of all it is possible that the altitude of Kathmandu is too low rsp. the oxygen partial pressure there is too high to show a significant energy depletion of the inner ear by the noise exposure of the study. However, this is not very probable since such an exposure is well known as putting ears at risk, a fact published in hundreds of papers and it has been also referred in the EU regulations (Anonymous 2003; Anonymous 2004), although, compared to other studies [e.g. 90 dB for 20 min (Oeken and Menz 1996)], we chose an exposure which was relatively low for ethical reasons.

Another argument against the hypothesis of the oxygen partial pressure at Kathmandu is that even at that altitude a TTS had been induced in our study. However, also the acclimatization process may be responsible for our observation: acclimatization includes numerous processes that all aim to increase the metabolic situation of cells in hypoxic environment as there are e.g. increased pulse rate, hyperventilation, increase of hemoglobin mass, higher capillary density, increased hypoxic ventilatory response, reduced hypoxic pulmonary pressure response etc. [survey in Ward et al. (2000), Kupper et al. (2010)]. Some mechanisms take effect within seconds (e.g. pulse rate) while some others need some 1000 years for genetical rsp. evolutionary reasons (e.g. pulmonary hypoxic pressure response). The so-called “ventilatory acclimatization”, which develops within about 10–20 days of permanent altitude exposure, is of special interest for our hypothesis that acclimatization may protect the inner ear (Kupper et al. 2010; Thomas 1991): after acute exposure to 3370 m the subjects in THOMAS’ study showed a mean decrease of arterial oxygen saturation from 96 to 88% followed by an increase to 91% after day 10 (Thomas 1991). Although this causes a significant effect on the aerobic capacity of humans at altitude, this is obviously not enough to ameliorate the metabolic deficiencies of the inner ear against TTS at altitude.

It might be discussed, that the time between two measurements was insufficient for a complete recovery of the inner hearing cells. Studies show, however, that the “most significant recovery was found to occur during the first 15 min following cessation of the noise exposure” (Ward 1970), the time necessary for complete recovery might be dose-dependent. Chen et al. published that after an exposition of 95 dB up to 2 h might be needed for recovery (2007) while Pierson found in 1971 that after 100 dB for a total of 8 h a complete recovery of the auditory system needed 48 h (1971). In our measurements, time of exposure was much shorter and the time between the measurements was several days for logistical reasons (hike to Ghorak Shep and back to Kathmandu). Therefore insufficient recovery time can be definitively excluded as biasing factor.

## Conclusions

The auditory system is more at risk for noise-induced damage in hypobaric hypoxia (at high altitude) than in normobaric normoxic conditions (at sea level). Our data could not prove a protective effect of acclimatization. Current regulations regarding thresholds of noise exposure which recommend that at > 80 dB ear protection should be provided and at > 85 dB it should be used (Anonymous 2003) are not sufficient for noise exposure at high altitude. Based on our data which indicate a moderate effect and taking into account that individuals react differently to altitude exposure and show significantly different levels of arterial oxygen saturation at the same altitude [survey in Ward et al. (2000), Kupper et al. (2010)] we suggest to use > 75 dB and > 80 dB as respective “red lines” to protect the ears in a noisy environment at altitudes above 1500 m. While intervals of 5 dB are well established in occupational health and safety the altitude corresponds to those which is commonly known as “threshold altitude” in high altitude medicine and physiology [survey in (Kupper et al. 2010)].
